# Clinical characteristics and survival outcomes in patients aged 75 years or older with advanced colorectal cancer treated using traditional Chinese medicine: an observational retrospective study

**DOI:** 10.3389/fphar.2023.1099659

**Published:** 2023-04-20

**Authors:** Jiao Wu, Ning Cui, Zhongyu Li, Yu Wu, Tengteng Hao, Liusheng Li

**Affiliations:** Oncology Department of Xiyuan Hospital, China Academy of Chinese Medical Sciences, Beijing, China

**Keywords:** advanced colorectal cancer, elderly, survival outcomes, clinical characteristics, traditional Chinese medicine

## Abstract

Limited evidence suggests that elderly patients with advanced colorectal cancer (ACRC) may benefit from traditional Chinese medicine (TCM). This study investigated the efficacy and safety of TCM in old ACRC patients treated in the Oncology Department of Xiyuan Hospital between January 2012 and December 2021. The clinical characteristics of these patients were retrospectively reviewed. Their progression-free survival (PFS) and total duration of TCM therapy (TTCM) were analyzed using the Kaplan-Meier curve. Forty-eight patients (F:M 13:35) with a mean age of 78.75 ± 2.99 years (range, 75–87) met the inclusion criteria. There were 18 cases of rectal cancer and 30 of colon cancer. The median PFS was 4 months (range, 1–26; 95% CI 3.26–4.73). The median TTCM was 5.5 months (range, 1–50; 95% CI 1.76–8.24). Subgroup analysis revealed that PFS and TTCM were shorter in patients with bone metastases and an ECOG performance status score of 2–3 (*p* < 0.05). No hematological toxicity or serious adverse reactions occurred during the study period. This real-world study demonstrates that TCM may be a potentially beneficial therapy for old ACRC patients, including when the ECOG performance status score is 2–3.

## 1 Introduction

Colorectal cancer (CRC) is one of the most common malignancies and has high morbidity and mortality rates (Song et al., 2020). In China, CRC ranks third among all cancers in terms of overall incidence, third for mortality in women, and fifth for mortality in men (Feng et al., 2019). CRC is the second leading cause of cancer-related death in patients aged 60–79 years and the third leading cause of death in patients aged 80 years and older (Hamed et al., 2022). However, regarding conventional medicine, there are still no guidelines for patients with advanced CRC (ACRC) who are aged ≥ 75 years because patients in this age group are usually excluded from clinical studies (Audisio and Papamichael, 2012). Nevertheless, some elderly patients with an Eastern Co-operative Oncology Group performance status (ECOG-PS) score of 0–1 may be suitable candidates for clinical studies in the Western medicine. Yet, the toxicity of the anti-cancer agents developed in the West is still greater in elderly patients than in their young counterparts, resulting in reduced quality of life and a shorter life expectancy (Shibutani et al., 2021).

Traditional Chinese medicine (TCM) has been used to treat various diseases for thousands of years in China. The history of treatment of CRC in China can be traced back thousands of years to a description of CRC and its treatment in the classic Chinese book “*Huangdi Neijing*” written 2,000 years ago. TCM is now covered by the medical insurance system in China and is very cost-effective. Therefore, it is readily accessible by the general population. Recent clinical studies have also confirmed that TCM has anti-tumor activity and prolongs survival without serious side effects (Sun et al., 2021). TCM can also improve quality of life, relieve symptoms, and reduce adverse events in patients with ACRC undergoing conventional chemotherapy (Zhang et al., 2018; Yan et al., 2021; Chen et al., 2022); Moreover, TCM can significantly prolong overall survival and progression-free survival (PFS) (Liu et al., 2020; Zhu et al., 2021). There are clinical cases reports of elderly patients with ACRC deriving benefit from TCM, with some taking TCM for up to 50 months. In view of these reports and the lack of data on TCM in elderly patients with CRC, the aim of our study was to explore the efficacy and safety of TCM in elderly patients with ACRC.

## 2 Materials and methods

### 2.1 Study design and participants

This observational retrospective study analyzed the clinical data, including for survival, in elderly patients who had been treated with TCM for ACRC in the Oncology Department of Xiyuan Hospital, China Academy of Chinese Medical Sciences between January 2012 and December 2021. The inclusion criteria are as follows: (Song et al., 2020): pathological diagnosis of CRC, (Feng et al., 2019), stage IV disease according to the National Comprehensive Cancer Network guidelines, and (Hamed et al., 2022) age ≥ 75 years. Study participants had received or were continuing to receive TCM whether or not they had a favorable response to conventional chemotherapy, including oxaliplatin, irinotecan, 5-fluorouracil, capecitabine, and anti-vascular endothelial growth factor and anti-epidermal growth factor receptor agents. The primary endpoint was PFS; secondary endpoints included the total duration of TCM (TTCM), disease control rate (DCR), and incidence of treatment-related adverse events (TRAEs).

### 2.2 Tumor assessment and TCM

Tumor size was assessed in each patient before treatment according to RECIST (Response Evaluation Criteria in Solid Tumors) version 1.1 by spiral computed tomography, which was performed at 3-month intervals from the start of treatment until disease progression or cessation of TCM. TCM is usually decoction that administered to strengthen the spleen and remove phlegm. Each prescription contains 16 natural herbs [*Astragalus* (*Leguminosae*), *Taizishen* (*Pseudostellaria*), *Epimedium* (*The Genus Epimedium*), *Tiannanxing* (*Arisaema*), *Ligustrum lucidum*, *Poria*, *Turmeric* (*Curcuma longa*), *ShiJianchuan* (*Labiatae Juss*)], administered twice a day, half an hour after breakfast and dinner. TCM can be administered as a decoction or as granules depending on the patient’s wishes. The TCM dose was adjusted according to each patient’s symptoms and tumor size or until the patient was no longer willing to take.

### 2.3 Data collection

Two investigators collected clinical information for all eligible patients from the outpatient records held at Xiyuan Hospital, Chinese Academy of Chinese Medical Sciences, including sex, age, location of the primary tumor, site(s) of metastasis, whether the primary site was treated surgically, post-visit treatments, ECOG-PS score, PFS, and TTCM.

### 2.4 Outcomes

The date of the last visit was assumed to be the time of progression if the patient discontinued treatment before the disease had progressed. PFS and TTCM were calculated from the day when treatment was started until disease progression for any reason, until the last dose of TCM, or until death from any cause. DCR is defined in RECIST version 1.1 as the objective response of the disease to an agent that has been administered for 6 months and includes complete response, partial response, stable disease, and progressive disease. TRAEs were assessed according to the Common Terminology Criteria for Adverse Events, version 4.0. The last follow-up was on 31 December 2021.

### 2.5 Statistical analysis

Patient basic characteristics and the incidence of TRAEs were analyzed descriptively. PFS and TTCM were analyzed by the Kaplan-Meier method. The 95% confidence interval (*CI*) was calculated. Statistical analyses were performed using SPSS version 25.0 software (IBM Corp., Armonk, NY, United States). A *p*-value <0.05 was considered statistically significant.

## 3 Results

### 3.1 Patient characteristics

Forty-eight patients (35 men, 13 women) aged ≥ 75 years with ACRC were eligible for inclusion in our study. The mean patient age was 78.75 ± 2.99 years (range, 75–87). There were 18 cases of rectal cancer and 30 of colon cancer. Thirty-eight patients underwent primary surgery (radical resection, *n* = 31; palliative surgery, *n* = 7) and 10 did not. Twenty-two patients received TCM alone and 26 received TCM in combination with conventional medicine. Six of the 48 patients progressed after first-line therapy, 10 were receiving first-line therapy, one was intolerant of first-line therapy and received only one cycle, four were receiving second-line therapy, and one was intolerant of second-line therapy and received only one cycle. Two patients were receiving targeted third-line therapy, one was undergoing radiotherapy for distant lymph node metastasis, one had just completed a course of radiotherapy, one was unable to complete radiotherapy because of a low platelet count, three had just undergone radio-frequency ablation for metastases, six had just been diagnosed to have advanced disease and not received conventional chemotherapy, and 12 had sought TCM because they were unwilling or unable to receive conventional therapy. Twenty patients had an ECOG-PS score of 1, 18 had a score of 2, and 10 had a score of 3. The patient characteristics are summarized in Table 1.

**TABLE 1:** Basic characteristics of patients at the time of initial treatment.

	*N* = 48	Percentage (%)
Age (total)	78.75 ± 2.99 (75–87)	
< 80 years old	30	62.5
≥ 80 years old	18	37.5
Gender		
Men	35	72.9
Women	13	27.1
Primary location		
Rectal	18	37.5
Colon	30	62.5
Differentiation		
Moderate	25	52.1
Poorly	12	25
Unknown	11	22.9
Tumor type		
Adenocarcinoma	35	72.9
Other	4	8.3
Unknown	9	18.8
Metastatic site		
Liver	28	58.3
Lung	14	29.2
Peritoneum	6	12.5
Bone	5	10.4
Distant lymph nodes	14	29.2
Single-organ metastasis	27	56.3
Multi-organ metastasis	21	43.8
Primary site surgery		
Radical surgery	31	64.6
Palliative surgery	7	14.6
Not operated	10	20.8
Treatment methods		
TCM	22	45.8
Integrative TCM and Western Medicine	26	54.2
ECOG-PS		
1	20	41.7
2	18	37.5
3	10	20.8

### 3.2 Survival outcomes

**FIGURE 1:**
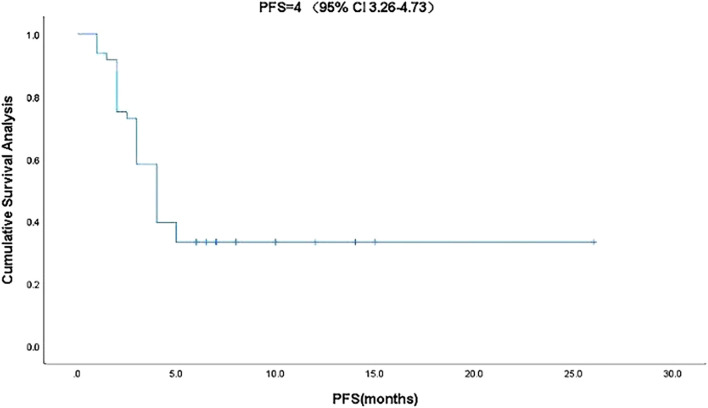
Kaplan-Meier plot showing PFS in patients with advanced colorectal cancer who received traditional Chinese medicine. CI, confidence interval; PFS, progression-free survival.

**FIGURE 2:**
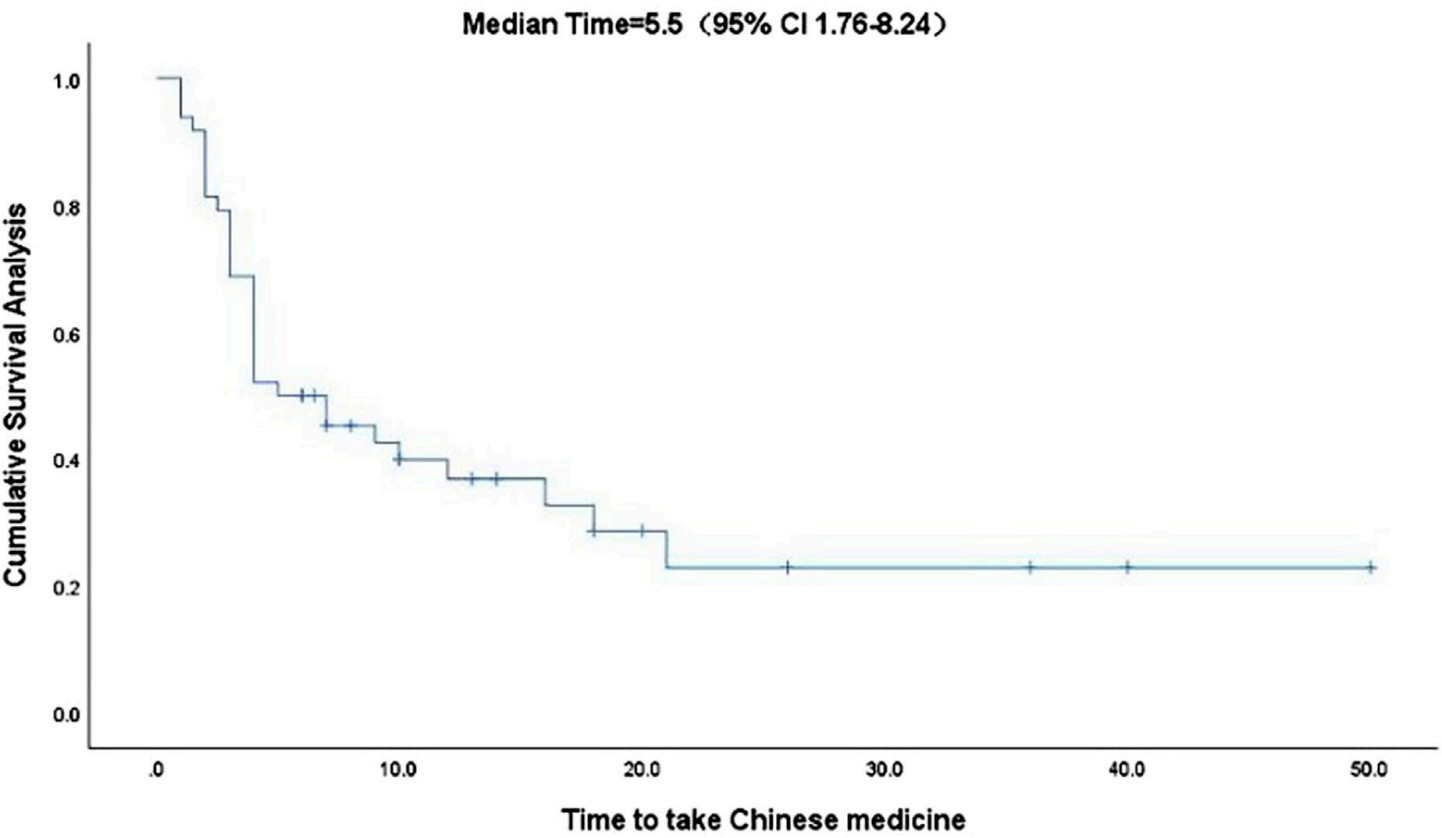
Kaplan-Meier plot showing the TTCM in patients with advanced colorectal cancer. CI, confidence interval; TTCM, total duration of traditional Chinese medicine therapy.

**FIGURE 3:**
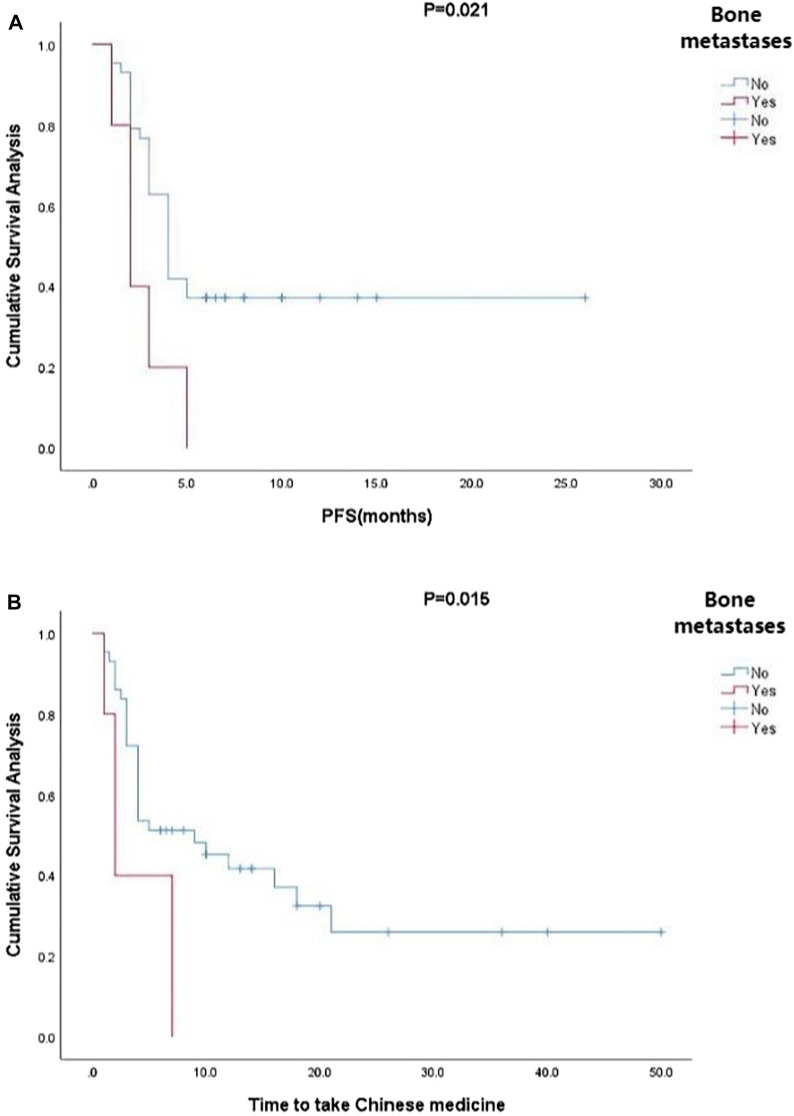
Kaplan-Meier plots showing **(A)** PFS and **(B)** TTCM in patients with advanced colorectal cancer and bone metastases. PFS, progression-free survival; TTCM, total duration of traditional Chinese medicine.

**FIGURE 4:**
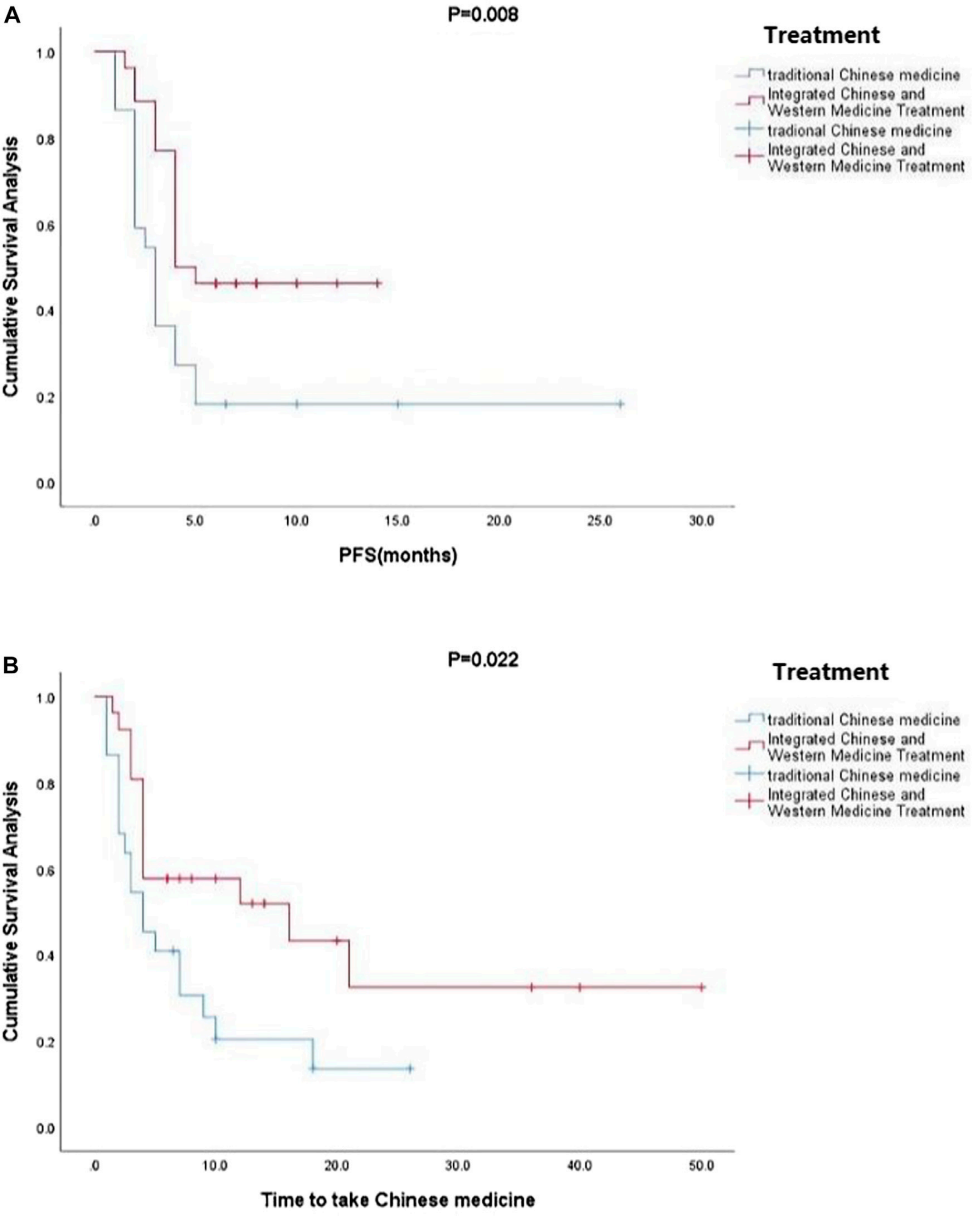
Kaplan-Meier plots showing **(A)** PFS and **(B)** TTCM according to whether or not TCM was combined with conventional Western chemotherapy. PFS, progression-free survival; TTCM, total duration of traditional Chinese medicine.

**FIGURE 5:**
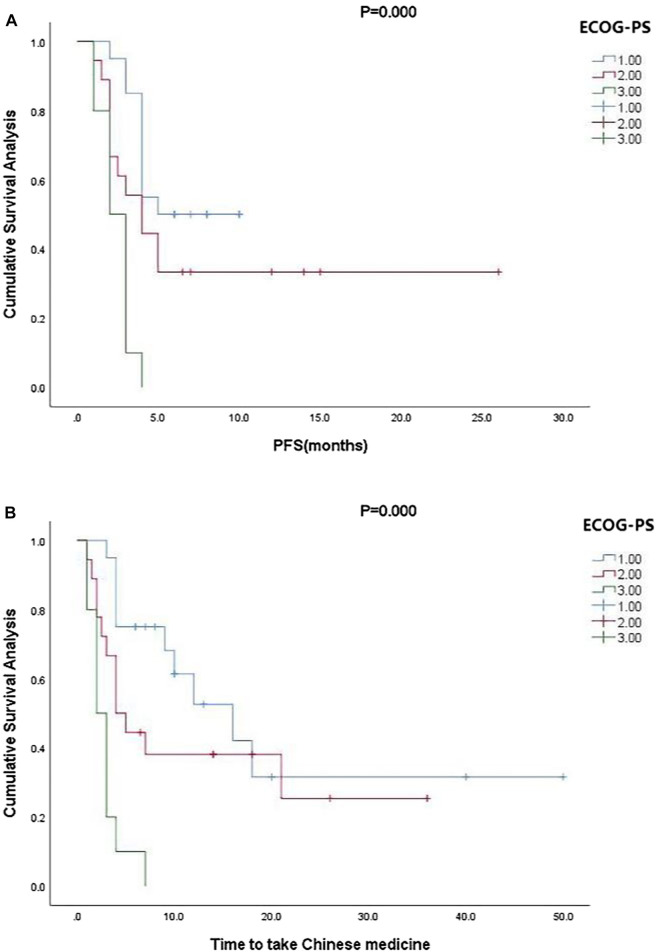
Kaplan-Meier plots showing **(A)** PFS and **(B)** TTCM according to ECOG-PS status. ECOG-PS, Eastern Cooperative Oncology Group performance status; PFS, progression-free survival; TTCM, total duration of traditional Chinese medicine.

Kaplan-Meier curve showed an overall median PFS of 4 months with a mean of 5.3 months (range, 1–26; 95% Cl 3.26–4.73; Figure 1) and a median TTCM of 5.5 months with a mean of 9.45 months (range, 1–50; 95% Cl 1.76–8.24; Figure 2). Subgroup analysis (Table 2) revealed that PFS and TTCM were significantly longer in patients with no bone metastasis, those who received a combination of TCM and traditional medicine, and those who had an ECOG-PS score of 1 than in patients with bone metastases, those who received only TCM, and those with an ECOG-PS score of 2–3 (*p* < 0.05; Figures 3–5).

**TABLE 2:** Subgroup analysis of PFS and TTCM.

	*N* = 48	Median PFS (months)	Median TTCM (months)	*p*-value (PFS)	*p*-value (TTCM)
Age (total)		4	5.5	0.390	0.945
< 80 years old	30	4	4		
≥ 80 years old	18	3	8.5		
Gender				0.819	0.958
Men	35	4	6.5		
Women	13	4	4		
Primary location				0.370	0.228
Rectal	18	4.5	8.5		
Colon	30	4	4		
Differentiation				0.418	0.151
Moderate	25	4	7		
Poorly	12	4	4		
Unknown	11	3	4		
Tumor type				0.333	0.113
Adenocarcinoma	35	4	6		
Other	4	7	11		
Unknown	9	3	4		
Metastatic site					
Liver	28	3.5	4	0.259	0.249
Lung	14	4.5	7.5	0.656	0.577
Peritoneum	6	4	5.5	0.713	0.700
Bone	5	2	6	**0.021**	**0.015**
Distant lymph nodes	14	4	6.25	0.987	0.64
Single-organ metastasis	27	4	5	0.488	0.343
Multi-organ metastasis	21	4	5	0.488	0.343
Primary site surgery				0.580	0.614
Radical surgery	31	4	7		
Palliative surgery	7	3	3		
Not operated	10	2.5	2.5		
Treatment methods				**0.008**	**0.022**
TCM	22	3	4		
Integrative TCM and Western Medicine	26	4.5	6.5		
ECOG-PS				**0.000**	**0.000**
1	20	5.5	9.5		
2	18	4	4.5		
3	10	2.5	2.5		

Note: Bold represents *p* < 0.05 or *p* < 0.1, which is statistically significant or potentially influential.

### 3.3 Disease control rate

After 6 months, the response to TCM was reassessed by spiral computed tomography. The response could not be assessed in 14 patients because they had been treated for up to 3 months without follow-up. No patient had a complete response, one had a partial response, 15 achieved stable disease, 18 had progressive disease, and 14 had disease that could not be evaluated (Table 3). The mean follow-up duration was 9.45 months (range, 1–50).

**TABLE 3:** Disease response rate.

	*n* (%)
Complete responses	0
Partial response	1 (2.1)
Stable disease	15 (31.3)
Progressive disease	18 (37.5)
Unevaluable	14 (29.2)

### 3.4 Safety analysis

Most patients were experienced slightly discomfort, such as fatigue, anorexia, diarrhea, abdominal distension and pain, constipation, and soreness around the abdomen and in the knees. Two patients developed diarrhea while taking TCM, and one developed constipation. Patients with advanced tumors had many symptoms, for which TCM could be ruled out as the cause. No relationship between was identified TCM and liver or kidney function. No obvious hematological toxicity or serious adverse reactions could be attributed to TCM during treatment.

## 4 Discussion

Currently, there’s no treatment guidelines exist for elderly patients with ACRC, mainly because patients aged ≥ 75 years are excluded from participation in clinical trials in the conventional medicine as a result of their high likelihood of comorbidities. Therefore, there is little information on treatment for ACRC and its efficacy in this age group. The limited literature available has focused on the short-term effects after surgery or on patients starting treatment at the age of 65–70 years (Cheng et al., 2022; Hashimoto et al., 2022), Or start at age 65 or 70 (Takahashi et al., 2021). Although there are some relevant clinical studies in patients aged ≥ 75 years, either the ECOG-PS score has been around 0–1 (Rosati et al., 2019), adverse events were severe (François et al., 2020), or there was only one metastatic site (Cuccia et al., 2021). Furthermore, most elderly participants in clinical research have undergone strict screening, requiring, for example, an ECOG-PS score of 0–1 or only one metastatic site for inclusion. Therefore, most clinical studies in the conventional Western medicine do not include patients aged ≥ 75 years with multiple metastases and an ECOG-PS score ≥ 2. Moreover, older adults may be more concerned about their quality of life and therapies that relieve their symptoms rather than receiving conventional curative Western chemotherapy, targeted therapy, or immunotherapy, which could have a high degree of toxicity and be burdensome for both patients and their families (Kemeny et al., 2003).

Patients treated with TCM do not need to be screened strictly for clinical research purposes. They can receive TCM provided that they can drink and are willing to do so. TCM is an independent factor affecting PFS (11); when combined with chemotherapy and cetuximab or bevacizumab, TCM has been shown to prolong PFS, improve quality of life, and reduce adverse reactions in patients with ACRC (11). Studies have shown that long-term use of TCM not only has a positive effect on survival in patients with CRC but also helps to reduce the risk of recurrence and metastasis (Wang et al., 2020). Pharmacological studies in animals or cell lines and network research have shown that TCM can prevent metastasis of CRC to the liver by down-regulating the activation of cancer-associated fibroblasts mediated by CRC-derived ITGBL1-loaded extracellular vesicles (Li et al., 2022); thus, TCM has the unique characteristics of being multi-targeted and multi-linked, and may have a comprehensive therapeutic effect. TCM can also inhibit epithelial–mesenchymal transition by downregulating trans-forming growth factor-beta, thereby inhibiting invasion and metastasis of CRC cells (Ge et al., 2022), inhibit progression of CRC by suppressing CCL2 and preserving progenitor Tex in an obese microenvironment (Xu et al., 2022) and suppress growth and metastasis of 5-fluorouracil-sensitive/resistant CRC by inhibiting the Wnt singling pathway (Zhang et al., 2022).

For the above reasons, this retrospective observational real-world study analyzed the clinical characteristics and survival data for patients aged ≥ 75 years with ACRC whose treatment included TCM. These patients had an overall median PFS of 4 months and a median TTCM of 5.5 months, which is in line with the PFS of 4.3 months reported for patients with wild-type KRAS metastatic CRC and frail older patients treated with panitumumab as a single agent in a study by the Spanish Digestive Oncology Collaborative Group (Sastre et al., 2015). However, the patients in that study were also strictly screened; the highest ECOG-PS score was 2% and 15.2% had adverse reactions that were grade ≥ 3. In contrast, our study included patients who did not undergo strict screening and was open to any patient who chose TCM and attended for follow-up. Our subgroup analysis found that patients without bone metastases had longer PFS and TTCM than those with bone metastases, which is also consistent with another recent report (Lavacchi et al., 2021). Due to the small sample size in our study, there were few patients with bone metastases; nevertheless, of the five patients with multiple bone metastases, four also had liver and lung metastases, pelvic metastases, and distant lymph node metastases. Therefore, no conclusions can be drawn regarding TCM and PFS in patients with CRC and bone metastases. Quality of life and survival in patients with bone metastases is markedly decreased due to pain, walking difficulties, pathological fractures, and neurological impairment (Rocha et al., 2022). Although TCM cannot reverse bone metastasis, it can have a positive effect on expression levels of markers of inflammation, apoptosis, and remission, thereby relieving pain and improving quality of life (Shen et al., 2022).

Studies have shown that patients with liver (Chuang et al., 2020) and peritoneal (Franko, 2018) metastases have a shorter survival time. However, our subgroup analysis found no significant difference in PFS or TTCM according to liver or peritoneal metastasis status. There are several possible explanations for these inconsistent findings. First, our sample size of 48 patients may have been too small to detect a statistically significant effect of TCM. Second, all our patients with liver metastases had metastases at multiple sites, including the lung, peritoneum, and distant lymph nodes. Similarly, patients with peritoneal metastasis also had metastases in the liver and abdominal and pelvic lymph nodes. Therefore, we cannot draw any conclusions regarding survival by simply analyzing liver and peritoneal metastases.

In our study, most of the patients who received TCM alone had an ECOG-PS score of 2–3 (*n* = 18) and most of those who received a combination of TCM and conventional anti-cancer treatment had an ECOG-PS score of 1 (*n* = 22). ECOG-PS is an important determinant of patient survival (LI, 2020), and also one of the important reasons for whether or not Western chemotherapy is recommended. With the exception of four patients who chose not to continue with conventional chemotherapy, those treated with TCM were not candidates for chemotherapy. Our subgroup analysis according to ECOG-PS score confirmed that the better the patient’s physical status, the longer the PFS and TTCM (Figure 5). This may be the main reason why PFS was shorter in our patients who were treated with TCM alone than in those treated with TCM in combination with conventional chemotherapy. Median PFS was 2.1 months in rigorously screened patients who received TAS-102 (Cicero et al., 2020). This value is similar to the median PFS of 3 months in our patients who received TCM alone, which had the additional advantages of low cost and fewer side effects. Overall, our research shows that TCM has advantages in treatment.

Surgery, radiotherapy, chemotherapy, targeted therapy, and immunotherapy remain the mainstay of treatment for CRC. However, for patients who have failed on or are ineligible for these treatments, TCM is their last chance for potentially helpful treatment. Most patients who choose TCM are in a poor physical state and seek TCM to alleviate the toxicity of conventional chemotherapy, have discontinued conventional anti-cancer treatments because of adverse reactions, or are not candidates for these therapies. All the patients in our study had multiple metastases, even if only one organ was involved. Furthermore, their ECOG-PS scores were worse than those in the clinical studies of Western anti-cancer treatments. More than half of our patients had an ECOG-PS score > 1. Many such patients attend for a medical consultation in a wheelchair, or are already bedridden, in which case their family members attend instead. However, even in these circumstances, the median PFS was still 4 months with a median TTCM of 5.5 months, which suggests that TCM is effective in prolonging survival and may improve quality of life.

This study has some advantages and limitations. The study found the potential benefits of TCM for elderly patients with ACRC who are physically weak and unable to receive conventional Western medicine treatment. In China, many elderly patients choose TCM treatment because they cannot tolerate conventional Western medicine treatment. In the course of clinical treatment and follow-up, we found that elderly patients with ACRC seem to benefit from TCM, but there is a lack of relevant data to prove a benefit of TCM for elderly patients with ACRC. Therefore, we carried out such a retrospective data summary analysis. Fortunately, we found a potential benefit of TCM in the elderly. TCM has a potential therapeutic value for the elderly patients with an ECOG score of 2-3 and who cannot tolerate conventional Western medicine treatment. Therefore, we would like to share this result in the hope that more studies can pay attention to this problem and better serve elderly patients.

However, there are still some shortcomings in this study. First, the study had a retrospective single-center design, which would have introduced a degree of bias. Second, although most elderly patients have comorbidities, these were not included in our analysis. Therefore, while some studies have shown that comorbidities have a marked impact on survival in the elderly (Canoui-Poitrine et al., 2022), we could not draw any conclusions in this regard. Third, the safety of TCM is also one of the topics of concern at present. Although no serious side effects of TCM were found in this study, the interaction between drugs should not be ignored. For this kind of study, we preliminarily found the potential benefits of TCM on elderly patients. However, due to the lack of rigor in design, more rigorous design, scientific statistical processing, strict inclusion of patients, and well follow-up information design is needed in future studies. Multicenter clinical studies in larger samples are required to determine the efficacy and safety of TCM in elderly patients with ACRC.

## 5 Conclusion

This retrospective observational real-world study suggests that TCM is potential effective in patients aged ≥ 75 years with advanced colorectal cancer. And TCM can be used safely in these patients, even if they have an ECOG-PS score of 2–3.

## Data Availability

The original contributions presented in the study are included in the article/supplementary material, further inquiries can be directed to the corresponding author.

## References

[B1] Audisio R. A. Papamichael D. (2012). Treatment of colorectal cancer in older patients. Nat. Rev. Gastroenterol. Hepatol. 9 (12), 716–725. 10.1038/nrgastro.2012.196 23045000

[B2] Canoui-Poitrine F. Segaux L. Benderra M. A. About F. Tournigand C. Laurent M. (2022). The prognostic value of eight comorbidity indices in older patients with cancer: The ELCAPA cohort study. Cancers (Basel). 14 (9), 2236. 10.3390/cancers14092236 35565364 PMC9105640

[B3] Chen D. Guo Y. Yang Y. (2022). Liujunanwei decoction attenuates cisplatin-induced nausea and vomiting in a Rat-Pica model partially mediated by modulating the gut micsrobiome. Front. Cell Infect. Microbiol. 12, 876781. 10.3389/fcimb.2022.876781 36061858 PMC9437319

[B4] Cheng Y. X. Liu X. Y. Kang B. Tao W. Wei Z. Q. Peng D. (2022). Comparison of surgical and oncologic outcomes in very elderly patients (≥80 years old) and elderly (65-79 years old) colorectal cancer patients: A propensity score matching. BMC Gastroenterol. 22 (1), 205. 10.1186/s12876-022-02277-y 35468733 PMC9036748

[B5] Chuang S. C. Huang C. W. Chen Y. T. Ma C. J. Tsai H. L. Chang T. K. (2020). Effect of KRAS and NRAS mutations on the prognosis of patients with synchronous metastatic colorectal cancer presenting with liver-only and lung-only metastases. Oncol. Lett. 20 (3), 2119–2130. 10.3892/ol.2020.11795 32782529 PMC7400335

[B6] Cicero G. Addeo R. De Luca R. Lo Re G. Gulotta L. Marchesa P. (2020). TAS-102 in metastatic colorectal cancer (mCRC): Efficacy, tolerability, and quality of life in heavily pretreated elderly patients: A real-life study. Drugs Context 9, 1–8. 10.7573/dic.2020-6-3 PMC7505119 32994802

[B7] Cuccia F. Mazzola R. Pastorello E. Figlia V. Giaj-Levra N. Nicosia L. (2021). SBRT for elderly oligometastatic patients as a feasible, safe and effective treatment opportunity. Clin. Exp. Metastasis 38 (5), 475–481. 10.1007/s10585-021-10122-x 34487288

[B8] Feng R. M. Zong Y. N. Cao S. M. Xu R. H. (2019). Current cancer situation in China: Good or bad news from the 2018 global cancer statistics? Cancer Commun. (Lond) 39 (1), 22. 10.1186/s40880-019-0368-6 31030667 PMC6487510

[B9] François E. Mineur L. Deplanque G. Laplaige P. Smith D. Gourgou S. (2020). Efficacy and safety of bevacizumab combined with first-line chemotherapy in elderly (≥75 Years) patients with metastatic colorectal cancer: A real-world study. Clin. Colorectal Cancer 19 (3), e100–e109. 10.1016/j.clcc.2020.02.009 32299778

[B10] Franko J. (2018). Therapeutic efficacy of systemic therapy for colorectal peritoneal carcinomatosis: Surgeon's perspective. Pleura Perit. 3 (1), 20180102. 10.1515/pp-2018-0102 PMC6405010 30911652

[B11] Ge H. Xu C. Chen H. Liu L. Zhang L. Wu C. (2022). Traditional Chinese medicines as effective reversals of epithelial-mesenchymal transition induced-metastasis of colorectal cancer: Molecular targets and mechanisms. Front. Pharmacol. 13, 842295. 10.3389/fphar.2022.842295 35308223 PMC8931761

[B12] Hamed R. A. Korpanty G. Kelly D. (2022). Toxicities and outcomes of neoadjuvant treatment in elderly patients with locally advanced rectal cancer: A scoping review protocol. BMJ Open 12 (5), e061397. 10.1136/bmjopen-2022-061397 PMC9062800 35501084

[B13] Hashimoto S. To K. Wada H. Sakakibara Y. Ozeki K. Komaki M. (2022). Total risk points predict short- and long-term outcomes following colorectal cancer resection in older patients. Cancer Diagn Progn. 2 (3), 360–368. 10.21873/cdp.10117 35530652 PMC9066536

[B14] Kemeny M. M. Peterson B. L. Kornblith A. B. Muss H. B. Wheeler J. Levine E. (2003). Barriers to clinical trial participation by older women with breast cancer. J. Clin. Oncol. 21 (12), 2268–2275. 10.1200/JCO.2003.09.124 12805325

[B15] Lavacchi D. Roviello G. Giommoni E. Dreoni L. Derio S. Brugia M. (2021). Aflibercept plus folfiri as second-line treatment for metastatic colorectal cancer: A single-institution real-life experience. Cancers (Basel) 13 (15), 3863. 10.3390/cancers13153863 34359764 PMC8345481

[B16] Li R. Zhou J. Wu X. Li H. Pu Y. Liu N. (2022). Jianpi Jiedu Recipe inhibits colorectal cancer liver metastasis via regulating ITGBL1-rich extracellular vesicles mediated activation of cancer-associated fibroblasts. Phytomedicine 100, 154082. 10.1016/j.phymed.2022.154082 35381565

[B17] Li Z. (2020). A retrospective study of advanced early-onset colorectal cancer and later-onset 384colorectal cancer [master]: Chinese Academy of traditional Chinese medicine.

[B18] Liu N. Wu C. Jia R. Cai G. Wang Y. Zhou L. (2020). Traditional Chinese medicine combined with chemotherapy and cetuximab or bevacizumab for metastatic colorectal cancer: A randomized, double-blind, placebo-controlled clinical trial. Front. Pharmacol. 11, 478. 10.3389/fphar.2020.00478 32372960 PMC7187887

[B19] Rocha M. M. Dariva I. Zornoff G. C. De Laurentis G. S. Mendes G. C. Santana M. G. (2022). A new therapeutic approach for bone metastasis in colorectal cancer: Intratumoral melittin. J. Venom. Anim. Toxins Incl. Trop. Dis. 28, e20210067. 10.1590/1678-9199-JVATITD-2021-0067 35321289 PMC8922758

[B20] Rosati G. Cordio S. Reggiardo G. Aprile G. Butera A. Avallone A. (2019). Oxaliplatin-based chemotherapy in patients with metastatic colorectal cancer aged at least 75 Years: A post-hoc subgroup analysis of three phase II trials. Cancers (Basel) 11 (4), 578. 10.3390/cancers11040578 31022922 PMC6521155

[B21] Sastre J. Massuti B. Pulido G. Guillén-Ponce C. Benavides M. Manzano J. L. (2015). First-line single-agent panitumumab in frail elderly patients with wild-type KRAS metastatic colorectal cancer and poor prognostic factors: A phase II study of the Spanish cooperative group for the treatment of digestive tumours. Eur. J. Cancer 51 (11), 1371–1380. 10.1016/j.ejca.2015.04.013 25963019

[B22] Shen Y. Wang J. Yan P. Chen T. Li X. Jiang M. (2022). The pharmacological mechanism of the effect of plant extract compound drugs on cancer pain based on network Pharmacology. J. Healthc. Eng. 2022, 9326373. 10.1155/2022/9326373 35265311 PMC8898871

[B23] Shibutani M. En W. Okazaki Y. Kashiwagi S. Fukuoka T. Iseki Y. (2021). The efficacy and safety of trifluridine/tipiracil treatment for elderly patients with metastatic colorectal cancer in a real-world setting. Anticancer Res. 41 (12), 6211–6216. 10.21873/anticanres.15440 34848475

[B24] Song C. Xu W. Wu H. Wang X. Gong Q. Liu C. (2020). Photodynamic therapy induces autophagy-mediated cell death in human colorectal cancer cells via activation of the ROS/JNK signaling pathway. Cell Death Dis. 11 (10), 938. 10.1038/s41419-020-03136-y 33130826 PMC7603522

[B25] Sun Q. He M. Zhang M. Zeng S. Chen L. Zhao H. (2021). Traditional Chinese medicine and colorectal cancer: Implications for drug discovery. Front. Pharmacol. 12, 685002. 10.3389/fphar.2021.685002 34276374 PMC8281679

[B26] Takahashi M. Sakamoto Y. Ohori H. Tsuji Y. Kuroki M. Kato S. (2021). Phase II study of trifluridine/tipiracil (TAS-102) therapy in elderly patients with colorectal cancer (T-CORE1401): Geriatric assessment tools and plasma drug concentrations as possible predictive biomarkers. Cancer Chemother. Pharmacol. 88 (3), 393–402. 10.1007/s00280-021-04277-3 34028598 PMC8316169

[B27] Wang Y. Liu P. Fang Y. Tian J. Li S. Xu J. (2020). The effect of long-term traditional Chinese medicine treatment on survival time of colorectal cancer based on propensity score matching: A retrospective cohort study. Evid. Based Complement. Altern. Med. 2020, 7023420. 10.1155/2020/7023420 PMC7013320 32089727

[B28] Xu Y. Wang H. Wang T. Chen C. Sun R. Yao W. (2022). Dahuang Fuzi Baijiang decoction restricts progenitor to terminally exhausted T cell differentiation in colorectal cancer. Cancer Sci. 113 (5), 1739–1751. 10.1111/cas.15311 35238098 PMC9128181

[B29] Yan S. H. Feng S. Xu Y. Yan Y. Z. He B. Sun L. Y. (2021). Effectiveness of herbal medicine for leukopenia/neutropenia induced by chemotherapy in adults with colorectal cancer: A systematic review and meta-analysis. Integr. Cancer Ther. 20, 15347354211021654. 10.1177/15347354211021654 34116595 PMC8202260

[B30] Zhang S. Shi L. Mao D. Peng W. Sheng C. Ding C. (2018). Use of jianpi jiedu herbs in patients with advanced colorectal cancer: A systematic review and meta-analysis. Evid. Based Complement. Altern. Med. 2018, 6180810. 10.1155/2018/6180810 PMC5830191 29619070

[B31] Zhang W. Peng C. Yan J. Chen P. Jiang C. Sang S. (2022). Sanguisorba officinalis L. suppresses 5-fluorouracil-sensitive and-resistant colorectal cancer growth and metastasis via inhibition of the Wnt/β-catenin pathway. Phytomedicine 94, 153844. 10.1016/j.phymed.2021.153844 34785413

[B32] Zhu Y. Yu J. Zhang K. Feng Y. Guo K. Sun L. (2021). Network Pharmacology analysis to explore the pharmacological mechanism of effective Chinese medicines in treating metastatic colorectal cancer using meta-analysis approach. Am. J. Chin. Med. 49 (8), 1839–1870. 10.1142/S0192415X21500877 34781857

